# Triazine-based porous organic polymers for reversible capture of iodine and utilization in antibacterial application

**DOI:** 10.1038/s41598-022-06671-0

**Published:** 2022-02-16

**Authors:** Anandhu Mohan, Mohammad H. Al-Sayah, Abdelrahman Ahmed, Oussama M. El-Kadri

**Affiliations:** Department of Biology, Chemistry, and Environmental Sciences, American University of Sharjah, PO Box 26666, Sharjah, United Arab Emirates

**Keywords:** Materials science, Polymers, Chemistry, Materials chemistry, Nanoscale materials, Pollution remediation

## Abstract

The capture and safe storage of radioactive iodine (^129^I or ^131^I) are of a compelling significance in the generation of nuclear energy and waste storage. Because of their physiochemical properties, Porous Organic Polymers (POPs) are considered to be one of the most sought classes of materials for iodine capture and storage. Herein, we report on the preparation and characterization of two triazine-based, nitrogen-rich, porous organic polymers, NRPOP-1 (SA_BET_ = 519 m^2^ g^−1^) and NRPOP-2 (SA_BET_ = 456 m^2^ g^−1^), by reacting 1,3,5-triazine-2,4,6-triamine or 1,4-bis-(2,4-diamino-1,3,5-triazine)-benzene with thieno[2,3-b]thiophene-2,5-dicarboxaldehyde, respectively, and their use in the capture of volatile iodine. NRPOP-1 and NRPOP-2 showed a high adsorption capacity of iodine vapor with an uptake of up to 317 wt % at 80 °C and 1 bar and adequate recyclability. The NRPOPs were also capable of removing up to 87% of iodine from 300 mg L^−1^ iodine-cyclohexane solution. Furthermore, the iodine-loaded polymers, I_2_@NRPOP-1 and I_2_@NRPOP-2, displayed good antibacterial activity against *Micrococcus luteus* (ML), *Escherichia coli* (EC), and *Pseudomonas aeruginosa* (PSA). The synergic functionality of these novel polymers makes them promising materials to the environment and public health.

## Introduction

To reduce the concentration of carbon dioxide (CO_2_) in the atmosphere, which is produced mainly by fossil fuel power plants, and meet the world energy demands, many countries have optioned for nuclear energy as an alternative to fossil fuels given its high energy density, minimal carbon footprints, and low working cost^1–3^. Despite such advantages, exhaust fumes from nuclear power plants contain a significant amount of radioactive species that include ^129^I and ^131^I, leading to major environmental and biological implications^3,4^. It has been projected by the IAEA that nuclear electricity capacity will almost double by 2050, and therefore, necessitates the need to develop highly efficient materials for the capture and storage of radioactive species^5–7^. Inorganic composite materials like silver-based zeolite have been widely used in radioactive iodine capture^6,7^. However, such materials suffer from low iodine uptake capacity due to their limited surface area and high cost. Therefore, the development of novel adsorbent materials for iodine capture is of significant importance.

Iodine is widely used as a powerful and effective disinfectant owing to its ability to destroy a wide range of living disease-causing bacteria^8,9^. Nevertheless, it is unsafe when directly applied to wounds owning to its instability and high volatility^10–12^. Recently, the use of polyvinylpyrrolidone (PVP) as an adsorbent for iodine has been reported. Such polymers can stabilize iodine through complexation and readily release it and hence, exterminating pathogenic microorganisms like bacteria, virus, and fungi^13,14^. The major disadvantages of these types of polymers are the low performance in water medium because of their high solubility and the somewhat complicated synthetic routes that limit their scale-up production^15,16^.

Porous organic polymers (POPs) have emerged as potential adsorbents for many pollutants including CO_2_, heavy metals, organic vapors, dyes, etc^17–22^. Constructed from light atoms (H, C, N, O), POPs often show excellent thermal and chemical stabilities, high porosity, and tuneable chemical functionality, and thus can be easily tailored toward targeted applications^23^. POPs have been reported to be effective absorbents for revisable iodine sequestration with varying uptake capacity, release, and recyclability; however, the reutilization of the encapsulated iodine for potential applications has not been explored. Our group is interested in the development of triazine-based, aminal-linked porous organic polymers because they offer an optimized environment for the capture and storage of guest molecules through the introduction of chemical functional units via cost effective, metal-free condensation reactions. Previously, we reported on the synthesis of a number of nitrogen-rich aminal-linked POPs (NRAPOPs) that displayed excellent iodine and CO_2_ capturing capacities^24,25^. We attributed the high I_2_ and CO_2_ capture to the simultaneous effect of the high content of heteroatoms, porosity, and rich-π-electron conjugated structures, which contribute to the enhancement of the adsorbate-adsorbent interactions^26,27^. In this study, we extended the NRAPOPs library by preparing two cost-effective-novel porous polymers, NRPOP-1 and NRPOP-2, through metal free-Schiff base polycondensation reaction of 1,3,5-triazine-2,4,6-triamine (melamine) or 1,4-bis-(2,4-diamino-1,3,5-triazine)-benzene with thieno[2,3-b]thiophene-2,5-dicarboxaldehyde. The obtained porous polymers exhibit high iodine vapor capturing capacity of up to 317 wt% and have over 87% iodine removal efficiency from solution. The effects of heteroatoms and phenyl rings on the chemical and structural properties of the polymers and their contribution to iodine adsorption are discussed. Aside from their excellent iodine adsorption, the iodine-loaded NRPOPs (I_2_@NRPOPs) showed good antibacterial effectiveness against a wide range of bacteria that included *Micrococcus luteus* (ML), *Escherichia coli* (EC), and *Pseudomonas aeruginosa* (PSA) through their release of iodine. To the best of our knowledge, NRPOP-1 and NRPOP-2, present the first examples of porous organic polymers to utilize the encapsulated iodine in the antibacterial application. Our study herein suggests that other classes of porous materials, such as metal organic frameworks (MOFs), would also be reliable adsorbents for the capture and reutilization of pollutants in the fields of public health and chemical industry.

## Experimental section

### Material

All solvents and starting materials were bought from commercial suppliers (Sigma-Aldrich, Acros Organics, and Frontier Scientifics) and used without further purification. 1,4-bis-(2,4-diamino-1,3,5-triazine)benzene was prepared according to a previously reported procedure^28^.

### Synthesis of NRPOP-1

NRPOP-1 was synthesized by reacting 0.350 g (1.78 mmol) of thieno[2,3-b]thiophene-2,5-dicarboxaldehyde with 0.150 g (1.19 mmol) of 1,3,5-triazine-2,4,6-triamine (melamine) in 14.8 mL of anhydrous dimethyl sulfoxide (DMSO) at 180 °C for 72 h under argon atmosphere. The mixture was filtered, and the obtained solid was washed with tetrahydrofuran (THF), methanol, and acetone and dried under vacuum overnight to give a yellow-colored powder in 76% yield.

### Synthesis of NRPOP-2

In a similar fashion to the preparation of NRPOP-1, NRPOP-2 was synthesized by reacting 0.350 g (1.78 mmol) of thieno[2,3-b]thiophene-2,5-dicarboxaldehyde with 0.264 g (0.891 mmol) of 1,4-bis-(2,4-diamino-1,3,5-triazine)-benzene in 13.5 mL of anhydrous DMSO to give a light yellow-colored powder in 73% yield.

### Iodine adsorption and release studies

The gravimetric measurements based on iodine adsorption experiments were carried out by the following procedure: 40 mg of each polymer (NRPOP-1 and NRPOP-2) was taken in separate 10 mL glass vials. After noting the initial weight, the vials were placed in a glass jar container having an excess amount of solid iodine. The glass jar was then sealed and heated at 353 K and 1.0 bar in an oven. After the iodine adsorption, the system was allowed to cool down to room temperature and weighed afterward. The iodine adsorption capacities for the NRPOPs were calculated by the following equation:

$$\frac{{{\text{m}}_{2} - {\text{m}}_{1} }}{{{\text{m}}_{1} }}*100\;{\text{wt}}\% ,$$

where m_1_ and m_2_ are masses of polymer before and after adsorption. The adsorption isotherm and capacity of the dissolved iodine were evaluated by placing 3 mg of fresh NRAPOPs in 0.3 g mL^−1^ iodine–cyclohexane solution. The progress of the iodine adsorption was checked by UV–Vis spectrometer. The iodine release experiments were carried out using ethanol as the solvent. For that, 50 mL of ethanol was added to a conical flask containing the 5 mg of the iodine-loaded NRPOPs, and the extent of iodine released was monitored by UV–Vis spectroscopy at specific time intervals.

### Antibacterial activity of the iodine captured polymers

The antibacterial activity of the I_2_@NRPOPs was assessed against three bacteria species: *Escherichia coli* (EC, MicroKwik Culture # 155065A), *Pseudomonas aeruginosa* (PSA, MicroKwik Culture # 155250A), and *Micrococcus luteus* (ML, MicroKwik Culture # 155155A) using agar diffusion test. The bacterial inoculum was uniformly spread using a sterile cotton swab on a sterile Petri dish agar. Then, a sample (1 mg) of the iodine-loaded polymers was placed in the center of the dish and it was covered by a sterile wet (15 microL of water) paper disk (5 mm in diameter). For the control experiments, a sample of the unloaded polymer was used. After incubation for 24 h at 37 °C for EC and PSA, and at 25 °C for ML, the diameter of the growth inhibition zone was measured to determine the efficiency of the NRPOPs. Tests were performed in triplicates.

### Instrumentation and methods

Surface area and pore size distribution of the synthesized material were measured by using nitrogen adsorption and desorption isotherm (Quantochrome analyzer at 77 K), which were calculated by using Braunauer-Emmett-Teller (BET) and the non-local density functional theory (NLDFT) methods, respectively. Fourier transform infrared spectra were recorded by Perkin-Elmer FT-IR spectrometer. Thermogravimetric analysis (TGA, Perkin-Elmer thermogravimetric analyzer) was carried out to check the thermal stability of the material at a heating rate of 10 °C min^−1^ in a temperature range of 30–800 °C under air atmosphere. Powder X-ray diffraction (PXRD) patterns were measured in 2θ range of 5–90° at a scanning speed of 5° min^−1^ in room temperature by using a Panalytical X’pert pro multipurpose diffractometer having a Cu Kα radiation. SEM images were recorded with high resolution scanning electron microscope (TESCAN-LMU SEM). Iodine solution concentrations were measured using a UV–Vis spectrometer (Shimadzu UV-1800 spectrometer) coupled with a quartz cuvette.

## Results and discussion

### Synthesis and characterization

**Scheme 1.**
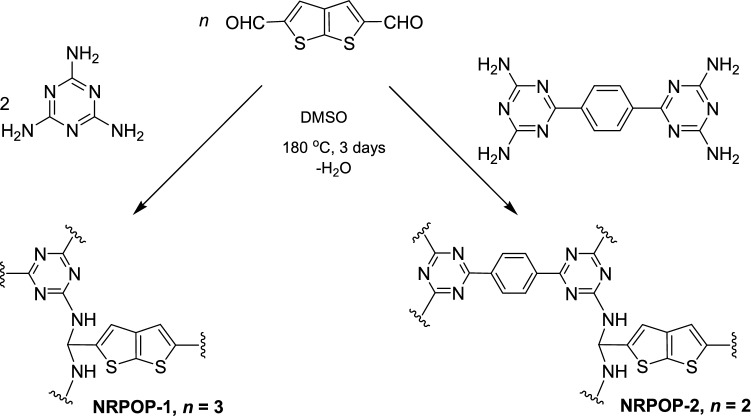
Schematic illustration of the synthesis of NRPOP-1 and NRPOP-2.

The triazine-based nitrogen-rich porous organic polymers (NRPOPs) were synthesized by Schiff-base reaction of 1,3,5-triazine-2,4,6-triamine (melamine) or 1,4-bis-(2,4-diamino-1,3,5-triazine)-benzene with thieno[2,3-b]thiophene-2,5-dicarboxaldehyde in DMSO at 180 °C for 72 h under argon atmosphere without the need of adding catalysts as depicted in Scheme 1. The obtained dark yellow (NRPOP-1) and light yellow (NRPOP-2) solids were characterized by spectroscopic and analytical techniques. The polymers are insoluble in common solvents, such as DMSO, dichloromethane (DCM), dimethylformamide (DMF), THF), chloroform (CHCl_3_), and ethanol (EtOH), suggesting a high degree of cross-linking, which facilitates their ease of isolation.

**Figure 1:**
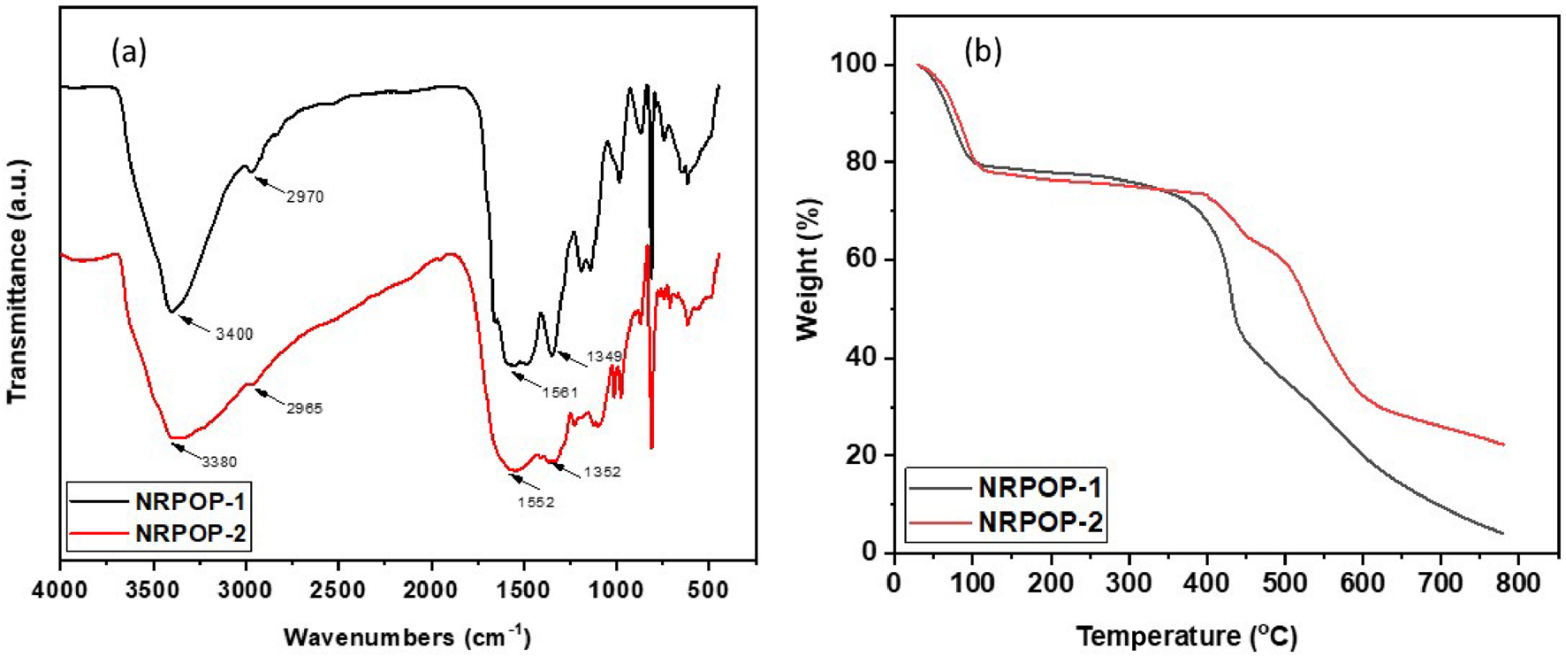
FTIR spectra (**a**) and TGA (**b**) of NRPOP-1 and NRPOP-2.

The successful formation of the NRPOPs was confirmed by FTIR spectroscopy (Fig. 1a). The aminal linkage formation between the triazine units and the aldehyde monomer is indicated by the appearance of stretching vibration bands at 3400/3380 and 2970/2965 cm^−1^ corresponding to secondary amine (N–H) and methylene (C–H) stretching. The inclusion of the triazine building block is confirmed by the presence of characteristic peaks at 1561/1552 and 1349/1352 cm^−1^^28,29^. In addition, the disappearance of the primary amine’s NH_2_ (stretching/deformation) peaks and the aldehyde’s stretching band of its C=O reveals the consumption of these groups which add another evidence for the formation of the NRPOPs (Fig. S1)^30,31^. The thermal stability of the as-prepared polymers was investigated by thermogravimetric analysis (TGA). Both NRPOPs show excellent thermal stability up to about 350 °C (Fig. 1b) because of the strong covalent interactions between the building blocks. The initial weight loss (below 100 °C) is presumably due to the evaporation of the guest molecules such as acetone and water^32^.

**Figure 2:**
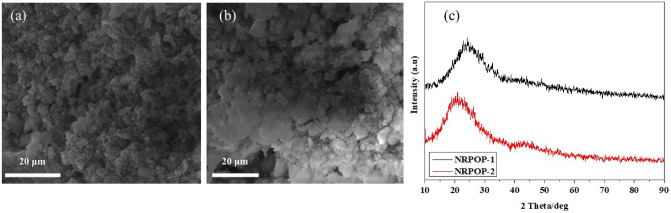
SEM images of NRPOP-1 (**a**) and NRPOP-2 (**b**) and their respective PXRD patterns (**c**).

The morphology of the synthesized samples was examined by scanning electron microscopy (SEM). Images of NRPOP-1 and NRPOP-2 (Fig. 2a,b) reveal that both polymers are composed of agglomerated spheres having different sizes. As the case of many POPs^24,25,33^, the two NRPOPs’ PXRD patterns confirmed their amorphous nature by showing characteristic broad peaks (Fig. 2c).

**Figure 3:**
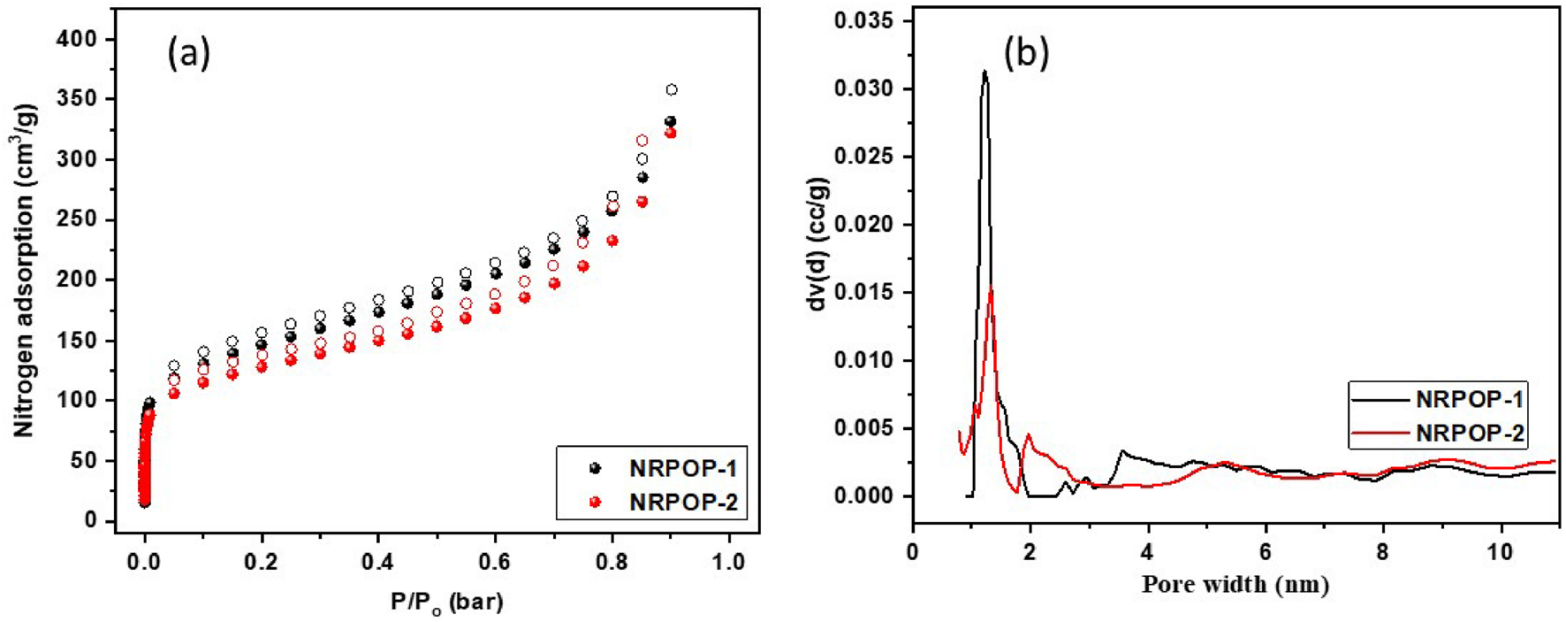
N_2_ adsorption–desorption isotherms (**a**) and pore size distribution curves (**b**) of NRPOP-1 and NRPOP-2.

Nitrogen adsorption–desorption experiments at 77 K were used to evaluate the porosity of the two POPs. As shown in Fig. 3a, the isotherms increase sharply at the lower pressure region (below 0.01 P/P_0_) which is indicative of the microporosity nature of the NRPOPs^34^. The reversible adsorption–desorption isotherms with minimal hysteresis suggest that the two NRPOPs are rigid presumably due to the highly cross-linked structures (Table 1 summarizes the textural properties of both polymers)^28,31^. The Brunauer-Emmet-Teller (BET) surface areas of NRPOP-1 and NRPOP-2 were determined to be 519 m^2^ g^−1^ and 456 m^2^ g^−1^, respectively. The lower surface area and the smaller total pore volume (0.675 cm^3^ g^−1^) of the NRPOP-2 polymer are presumably due to the existence of the phenyl group in the 1,4-bis-(2,4-diamino-1,3,5-triazine)benzene building block, leading to conformational flexibility in the polymer^3,33^. The pore size distribution (PSD) of the polymers were determined using the nonlocal-density-functional theory (NLDFT). The highest peaks were observed at 1.2 (NRPOP-1) and 1.3 nm (NRPOP-2) as presented in Fig. 3b.

**Table 1:** Textural properties of NRPOP-1 and NRPOP-2.

Sample	S_BET_ (m^2^ g^−1^)^a^	Pore volume (cm^3^ g^−1^)^b^	Pore size (nm)^c^
NRPOP-1	519	0.703	1.2
NRPOP-2	456	0.675	1.3

^a^BET surface area.

^b^Total pore volume at *P*/*P*_o_ = 0.95.

^*c*^Dominant pore size distribution calculated by NLDFT.

### Iodine capture and adsorption of the NRPOPs

**Figure 4:**
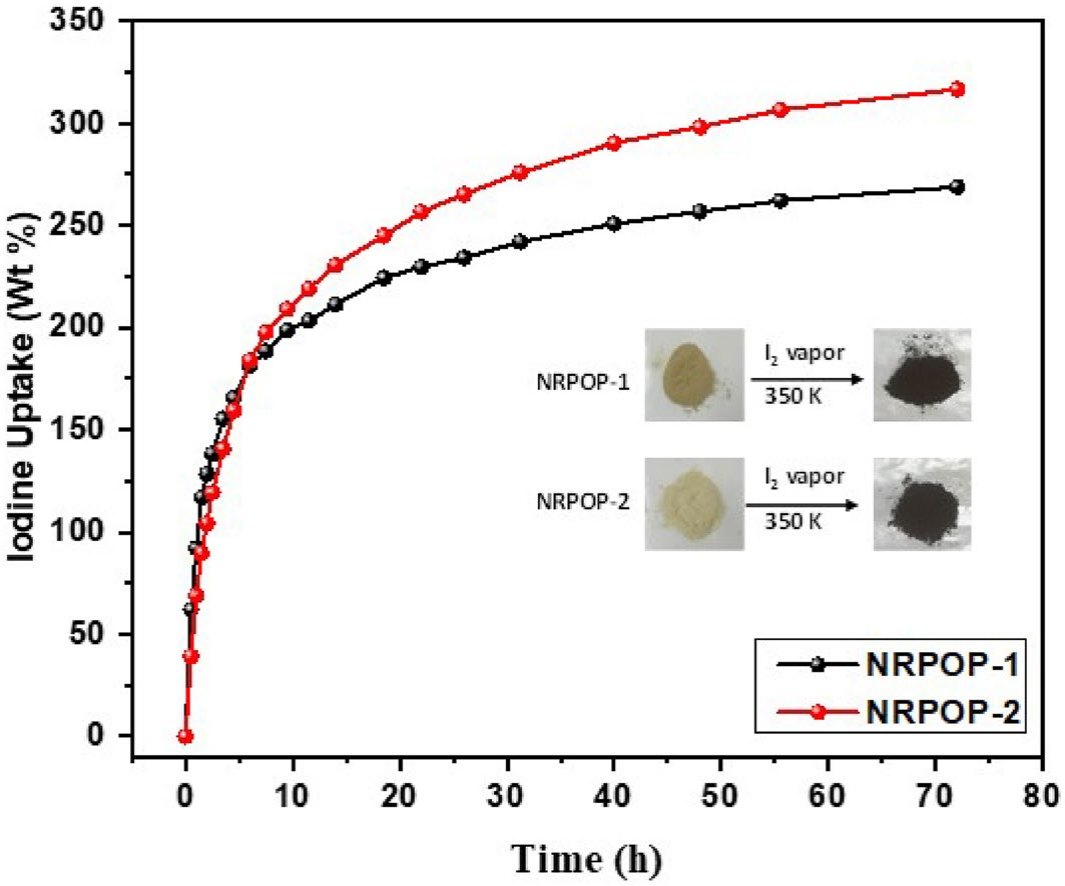
Gravimetric iodine uptake by NRPOPs overtime at 80 ℃ and ambient pressure; inset: photographs show the color change of NRPOPs after the exposure with iodine vapor.

The iodine capturing capacity of the NRPOPs was tested by adding known amounts of the NRPOPs into glass vials and placing them into a sealed glass jar containing an excess amount of iodine crystals. The system was heated up to 350 K and 1 bar and the samples weights were measured after some time intervals. In the first 8 h, both materials exhibited a significant increase in iodine adsorption (~ 200 wt%) presumably due to the vast abundance of adsorbing sites with an apparent color change from yellow to black (Fig. 4 inset). Afterward, the loading capacity continued to increase but at slower rates and reached equilibrium after 72 h of contact time with maximum iodine uptakes of 269 and 317 wt% for NRPOP-1 and NRPOP-2, respectively (Fig. 4). Such capacities are either higher or comparable to other porous aminal-linked porous polymers (Table S1). The obtained high iodine capacities of the two NRPOPs can be attributed to the presence of the electron deficient triazine moieties and –NH– unites which enhance the dipole–dipole interaction between the polymers structures and the iodine. In addition, the synergetic functionalities of the NRPOPs having both π-conjugated surfaces and rich-heteroatoms (N and S) provide another important favourable interaction between the adsorbates and the adsorbents^35,36^. The higher iodine uptake by NRPOP-2 can be attributed to the extra phenyl ring in its structure. Previous studies have shown that porous polymers bearing a plentiful number of phenyl rings enhance their affinity to adsorbates because they provide an electron-rich environment that enables stronger iodine adsorbent interactions^3,25,27,37^.

The iodine loading capacities were also studied by the TGA of the iodine loaded (I_2_@NRPOPs). The I_2_-NRPOPs curves show steep weight loss in the temperature range of 90 to 350 °C (Fig. S2). Such weight loss is presumably due to the release of the encapsulated iodine since the pristine polymers are stable under this temperature range. The weight loss of the iodine was estimated to be 2.65 and 2.71 g g^−1^ for I_2_@NRPOP-1 and I_2_@NRPOP-2, respectively, which are close to the equilibrium adsorption values especially for that of NRPOP-1. The difference in the weight loss and the saturated value of the NRPOP-2 is probably due to the incomplete release of the captured iodine due to its stronger interaction with the polymer as reasoned above. Major peaks in the IR spectra of the I_2_@NRPOPs revealed a significant shift compared to NRPOPs (Fig. S3). For example, the secondary amine bands of the I_2_@NRPOP-1 and I_2_@NRPOP-2 are red shifted by 84 and 60 cm^−1^, while the characteristic peaks of the triazine blue shifted by 49 and 66 cm^−1^, respectively. Such observations clearly suggest that the iodine is being captured at the secondary amine, aromatic rings, and triazine units of the NRPOPs. Similar to these shifts in the IR spectra have been observed in the iodine loaded HCMP-3 and iodine-doped PANi, and were attributed to the formation of charge transfer (CT) complexes between the polymers and polyiodide ions^38,39^. PXRD measurements of the I_2_@NRPOPs showed only broad peaks indicating their amorphous nature and absence of pristine iodine crystals (Fig. S4) and hence suggesting the iodine is encapsulated inside the pores.

**Figure 5:**
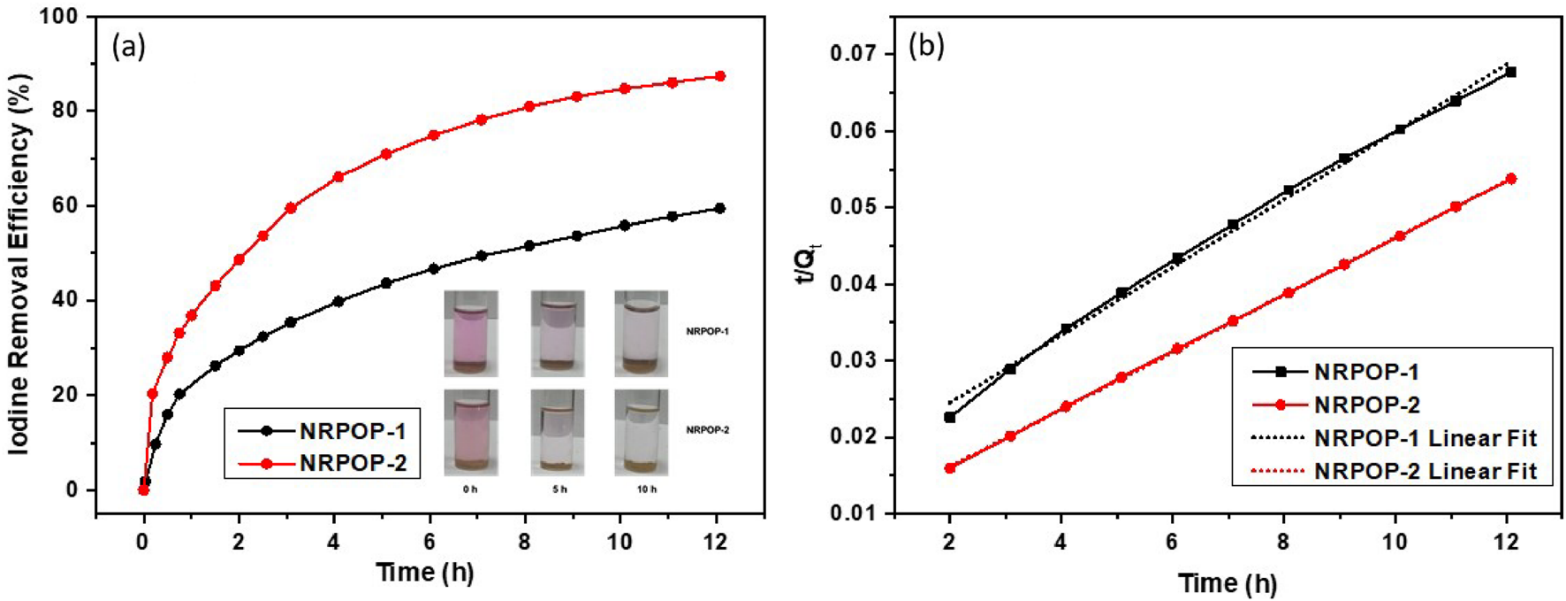
Iodine percentage removal from 300 mg L^−1^ iodine solution of cyclohexane by NRPOPs; Inset: photographs showing solutions color change after immersing NRPOPs into the solutions (**a**); pseudo-second-order kinetic fitting for iodine adsorption experimental data (**b**).

In addition to the gravimetric iodine vapor uptake, the ability of the synthesized polymers to adsorb iodine from the solution was investigated. In a typical experiment, 3 mg of the NRPOPs were immersed into 300 mg L^−1^ cyclohexane solutions of iodine (3 mL). With time, the color of the solutions gradually faded away from purple to light pink/colorless, indicating the removal of iodine from the solutions (Fig. 5a, inset). UV–Vis spectroscopic measurements were carried out to quantify the amount of the removed iodine and to monitor the progress of the adsorption rates. Figure 5a displays a plot of iodine percentage removal vs. time that clearly shows that the adsorption kinetics consists of two stages: (1) a rapid initial increase in the adsorptions in the first 2 h and (2) a slow steady increase afterward until equilibrium was reached. NRPOP-1 and NRPOP-2 exhibited an iodine removal efficiency of 59% (177 mg g^−1^ uptake) and 87% (261 mg g^−1^ uptake) from 300 mg L^−1^ cyclohexane solutions, respectively. The lower iodine uptake compared to that in the vapor phase is presumably due to the encapsulation of solvent molecules inside the NRPOPs pores. The faster and higher iodine removal efficiency of the NRPOP-2 can be related to the richer conjugated structure because of the presence of phenyl rings in its structure, and hence, leading to an enhanced iodine-host polymer interactions^3,40^. Recently, theoretical calculation (DFT) for iodine uptake studies have been reported that substantiate the experimental results^41^.

The obtained kinetics results were fitted into pseudo-first and pseudo-second-order kinetic equations (SI). The calculated *R*^2^ values (Table S2) indicate that the iodine adsorption kinetics fit better a pseudo-second-order kinetic model for both NRPOPs (Figs. 5b, S5). Such results suggest that the adsorption of iodine is governed by the chemisorption process^39,40^. This is attributed to the presence of high interaction between the iodine molecules and the surface of the NRPOPs. The presence of aromatic rings and electron-rich heteroatoms in the NRPOPs provide electron-donating moieties that are proficient in donating electrons to the electron-accepting iodine molecules, leading to charge transfer (CT) interactions and thus promoting higher iodine capturing capacity^3,24^. Previous reports indicated that such CT interactions are enhanced by the existence of –NH– units in the host structure^25,40^. This could be an additional factor for the excellent iodine uptake given the abundant –NH–containing pores of the NRPOPs.

**Figure 6:**
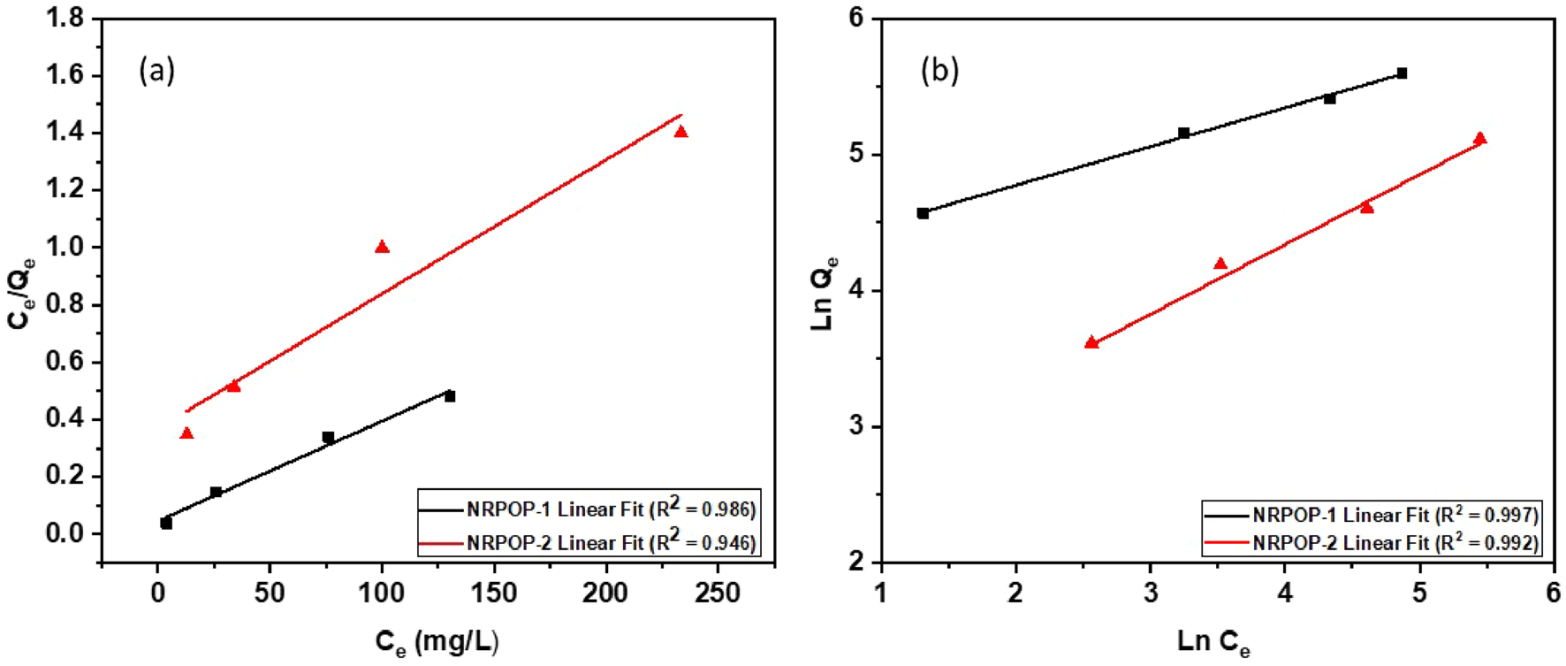
Iodine adsorption by NRPOPs isotherms fitted to Langmuir model (**a**) and Freundlich model (**b**). *R*^2^ is the correlation coefficient of the linear fit.

To gain more insight into the adsorption mechanism of the iodine by the NRPOPs, we carried out adsorption equilibrium experiments by adding 1 mg of each NRPOPs into a range of 1 mL iodine solutions of various concentrations at 298 K. After 72 h of soaking, the residual concentrations of iodine in the solutions were determined by UV– Vis spectroscopy. The obtained isotherm data were then fitted into the two widely utilized isotherm models: Langmuir and Freundlich (Fig. 6a,b). The Langmuir model suggests a monolayer adsorption on the surface with homogenous distribution in the active sites, whereas the Freundlich model suggests a reversible heterogeneous multilayer interaction^24,25^. The extracted isotherm parameters and linear regression coefficients are displayed in Table 2. The higher *R*^*2*^ values for the Freundlich model indicate that the equilibrium adsorption isotherms can be well fitted using this model. Accordingly, the adsorption of the iodine by NRPOPs can be described as a heterogeneous multilayer reversible adsorption process. Such conclusion is supported by the presence of different iodine adsorption sites within the NRPOPs that include triazine, dithiophene, and –NH– units^3^.

**Table 2:** Langmuir and Freundlich parameters for the adsorption of Iodine.

	Langmuir			Freundlich		
	Q_m_	K_l_	R^2^	N	K_f_	R^2^
NRPOP-1	285.7	0.749	0.986	3.52	67.10	0.997
NRPOP-2	212.8	0.0127	0.946	1.99	10.54	0.992

Langmiur model: Q_m_ = maximum monolayer coverage capacity (mg g^−1^), K_l_ = Langmuir isotherm constant (L mg^−1^); Freundlich model: K_f_ = Freundlich isotherm constant (mg g^−1^), N = adsorption intensity; *R*^2^ is the correlation coefficient of the linear fit.

### Iodine release from iodine-loaded NRPOPs and potential applications

**Figure 7:**
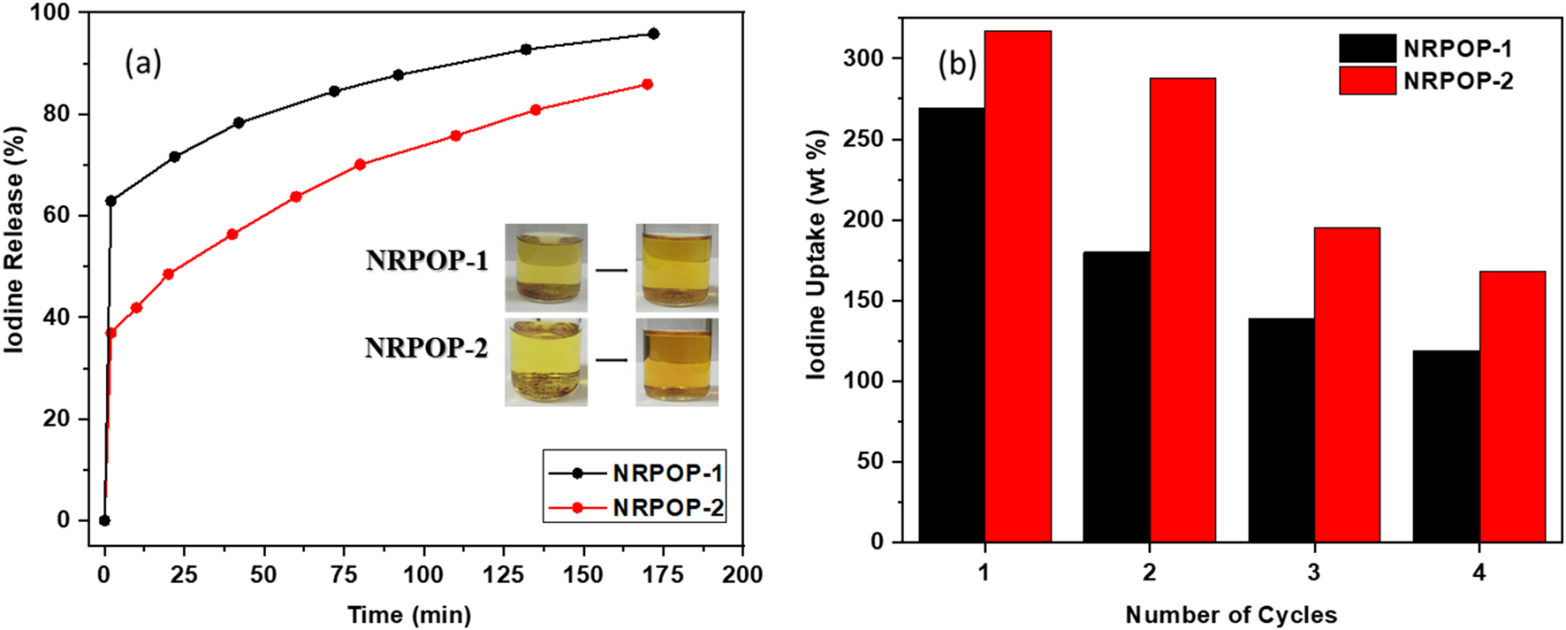
Percentage of iodine released from the NRPOPs by immersion in ethanol (**a**) and recyclability of NRPOPs for iodine vapor uptake (b).

The encapsulated iodine can be readily released upon placing the I_2_@NRPOPs in polar organic solvents such as ethanol at room temperature. To evaluate the releasing process, 5 mg of the I_2_@NRPOPs were immersed in ethanol (50 mL) as time progressed, the color of the solution gradually changed from colourless to brown indicating the release of the encapsulated iodine (Fig. 7a inset). The amount of iodine released into the ethanol solution was monitored by UV–vis measurements (Fig. 7a) at different time intervals. I_2_@NRPOP-1 and I_2_@NRPOP-2 exhibited a linear and quick release of iodine in the first 2 min followed by slow but steady increase over almost 3 h to reach a maximum iodine release efficiency of 95.8 and 85.9%, respectively. The linear initial increase implies pseudo-zero-order kinetics which is inductive of host–guest interactions^37^. It is interesting to note that the slower and lower iodine release efficiency of I_2_@NRPOP-2 is consistent with the higher iodine capture because of the strong π-conjugation interactions which facilitate the iodine capture, but also reduce the release efficiency and prolong the release time.

Considering the efficient release of iodine by the loaded polymers, this feature of I_2_@NRPOPs was further investigated for two potential applications. For the first application, the release of iodine from the polymers allows for another cycle of iodine loading which enables the compounds to act as transport carriers to move iodine from one medium to another. For example, NRPOPs can be used to remove pollutant radioactive iodine from the environment and release it into controlled containers with subsequent reuse of the polymers for more cycles. For the second application, the iodine-loaded polymers can be used to deliver iodine into a specific environment with controlled quantities. For example, since iodine is known to have antiseptic properties effective against a broad range of both gram-positive and negative bacteria^42,43^, I_2_@NRPOPs can be used as a delivery vehicle of iodine in such areas as wound dressing or bandage to prevent bacterial infections.

Accordingly, we investigated the reusability of the polymers by first soaking samples of I_2_@NRPOPs in ethanol to release the iodine and then applied thermal activation to release solvent molecules from the pores of the polymers. The regenerated samples were then subjected to gravimetric iodine uptake experiments (as described above). The NRPOP-1 and NRPOP-2 were able to capture up to 119 and 168 g g^−1^ of iodine, respectively, which correspond to 44 and 53% retention of their initial iodine uptake capacity (Fig. 7b) after four loading-release-regeneration cycles. The decrease in the loading efficiency is probably due to the strong iodine-polymers interactions, causing the incomplete release of the encapsulated time. The recycled NRPOPs were characterized further with PXRD and FTIR spectroscopy (Figs. S6 and S7). The results show the excellent stability and robustness of the POPs. The small shift toward the right side in the PXRD peaks after the fourth cycle is probably due to the incomplete release of the captured iodine by the stronger interaction between the polymer and iodine molecules.

### Antibacterial activity of the captured iodine

In order to investigate the potential application of I_2_@NRPOPs as a delivery vehicle of iodine for antiseptic action, we tested the ability of iodine-loaded NRPOPs to inhibit the growth of three types of bacteria, *Escherichia coli* (EC), *Pseudomonas aeruginosa* (PSA), and *Micrococcus luteus* (ML) using agar diffusion method. A sample (1 mg) of each iodine-loaded polymer was placed in the center of an agar petri dish seeded with the specific bacterial stain and the sample was covered by a wet paper disk. After incubation for 24 h, the diameter of the growth-inhibited zone was measured and compared to the control samples (polymers without iodine). The results presented in Table 3 (Fig. S8) reveal that both iodine-loaded NRPOP-1 and NRPOP-2 inhibited the growth of gram-negative bacteria, PSA and EC, and gram-positive bacteria ML with similar extents while the polymer alone (control) had no effect on bacterial growth. These results suggest that a significant amount of loaded iodine that was released from the polymer during the incubation time (24 h) prevented the growth of bacteria^44^, hence supporting the idea of potential application of NRPOPs as delivery vehicles of iodine for antiseptic applications.

**Table 3:** Diameters (cm) of inhibition rings of the bacteria on a petri dish by the loaded polymers.

	Control	EC^a^	PSA^b^	ML^c^
NRPOP-1	0	0.9 ± 0.1	1.8 ± 0.1	2.7 ± 0.2
NRPOP-2	0	0.9 ± 0.1	1.8 ± 0.1	2.0 ± 0.1

^a^*Escherichia coli* (MicroKwik Culture # 155065A).

^b^*Pseudomonas aeruginosa* (MicroKwik Culture # 155250A).

^c^*Micrococcus luteus* (MicroKwik Culture # 155155A).

## Conclusion

In summary, we synthesized two nitrogen rich aminal-linked porous organic polymers, NRPOP-1 and NRPOP-2, by metal free-Schiff base polycondensation reaction of 1,3,5-triazine-2,4,6-triamine (melamine) and 1,4-bis-(2,4-diamino-1,3,5-triazine)-benzene with thieno[2,3-b]thiophene-2,5-dicarboxaldehyde to attain the synergism of iodine uptake and the successive antibacterial use of the adsorbed iodine. Both polymers displayed excellent iodine vapor capturing capacity and were able to remove over 87% of dissolved iodine from 300 mg L^−1^ solution. The presence of phenyl units in the NRPOP-2 structure proved to be a major factor for the higher iodine uptake. The iodine-loaded polymers exhibited good antibacterial activity against the *Micrococcus luteus* (ML), *Escherichia coli* (EC), and *Pseudomonas aeruginosa* (PSA). The excellent iodine uptake and the effectiveness as antibacterial agents, NRPOPs make them promising candidates for environmental remediation and drug-delivery applications.

## Supplementary Information

Supplementary Information.

## References

[1] Cicia G, Cembalo L, Del Giudice T, Palladino A (2012). Fossil energy versus nuclear, wind, solar and agricultural biomass: Insights from an Italian national survey. Energy Policy.

[2] Rabilloud X (2013). Comments on ‘prevented mortality and greenhouse gas emissions from historical and projected nuclear power’. Environ. Sci. Technol..

[3] Abdelmoaty YH, Tessema TD, Choudhury FA, El-Kadri OM, El-Kaderi HM (2018). Nitrogen-rich porous polymers for carbon dioxide and iodine sequestration for environmental remediation. ACS Appl. Mater. Interfaces.

[4] Xie W, Cui D, Zhang SR, Xu YH, Jiang DL (2019). Iodine capture in porous organic polymers and metal-organic frameworks materials. Mater. Horizons.

[5] Alsbaiee A (2016). Rapid removal of organic micropollutants from water by a porous β-cyclodextrin polymer. Nature.

[6] Chapman KW, Chupas PJ, Nenoff TM (2010). Radioactive iodine capture in silver-containing mordenites through nanoscale silver iodide formation. J. Am. Chem. Soc..

[7] Subrahmanyam KS (2015). Chalcogenide aerogels as sorbents for radioactive iodine. Chem. Mater..

[8] Hetzel BS, Dunn JT (1989). The iodine deficiency disorders: Their nature and prevention. Annu. Rev. Nutr..

[9] Chen Y (2016). Preparation, property of the complex of carboxymethyl chitosan grafted copolymer with iodine and application of it in cervical antibacterial biomembrane. Mater. Sci. Eng. C.

[10] Bürgi H (2010). Iodine excess. Best Pract. Res. Clin. Endocrinol. Metab..

[11] Atwater JE, Sauer RL, Schultz JR (1996). Numerical simulation of iodine speciation in relation to water disinfection aboard manned spacecraft I. Equilibria. J. Environ. Sci. Health Part A Toxic Hazard. Subst. Environ. Eng..

[12] Lamme EN, Gustafsson TO, Middelkoop E (1998). Cadexomer-iodine ointment shows stimulation of epidermal regeneration in experimental full-thickness wounds. Arch. Dermatol. Res..

[13] Goodwin MJ (2017). Halogen and hydrogen bonding in povidone-iodine and related co-phases. Cryst. Growth Des..

[14] Sriwilaijaroen N (2009). Mechanisms of the action of povidone-iodine against human and avian influenza A viruses: Its effects on hemagglutination and sialidase activities. Virol. J..

[15] Papadopoulou EL (2018). Antibacterial bioelastomers with sustained povidone-iodine release. Chem. Eng. J..

[16] Gao B, Wang Z, Liu Q, Du R (2010). Immobilization of povidone-iodine on surfaces of silica gel particles and bactericidal property. Colloids Surf. B Biointerfaces.

[17] Das S, Heasman P, Ben T, Qiu S (2017). Porous organic materials: Strategic design and structure-function correlation. Chem. Rev..

[18] Rabbani MG, Sekizkardes AK, El-Kadri OM, Kaafarani BR, El-Kaderi HM (2012). Pyrene-directed growth of nanoporous benzimidazole-linked nanofibers and their application to selective CO_2_ capture and separation. J. Mater. Chem..

[19] Qian X (2017). Novel N-rich porous organic polymers with extremely high uptake for capture and reversible storage of volatile iodine. J. Hazard. Mater..

[20] Shen Y, Ni WX, Li B (2021). Porous organic polymer synthesized by green diazo-coupling reaction for adsorptive removal of methylene blue. ACS Omega.

[21] Huang L (2021). Nanoarchitectured porous organic polymers and their environmental applications for removal of toxic metal ions. Chem. Eng. J..

[22] Li G, Yao C, Wang J, Xu Y (2017). Synthesis of tunable porosity of fluorine-enriched porous organic polymer materials with excellent CO_2_, CH_4_ and iodine adsorption. Sci. Rep..

[23] Xu Y, Jin S, Xu H, Nagai A, Jiang D (2013). Conjugated microporous polymers: Design, synthesis and application. Chem. Soc. Rev..

[24] Sabri MA, Al-Sayah MH, Sen S, Ibrahim TH, El-Kadri OM (2020). Fluorescent aminal linked porous organic polymer for reversible iodine capture and sensing. Sci. Rep..

[25] Sen S, Al-Sayah MH, Mohammed MS, Abu-Abdoun II, El-Kadri OM (2020). Multifunctional nitrogen-rich aminal-linked luminescent porous organic polymers for iodine enrichment and selective detection of Fe^3+^ ions. J. Mater. Sci..

[26] Ren F (2016). Novel thiophene-bearing conjugated microporous polymer honeycomb-like porous spheres with ultrahigh iodine uptake. Chem. Commun..

[27] Jiang Q, Huang H, Tang Y, Zhang Y, Zhong C (2018). Highly porous covalent triazine frameworks for reversible iodine capture and efficient removal of dye. Ind. Eng. Chem. Res..

[28] Song WC, Xu XK, Chen Q, Zhuang ZZ, Bu XH (2013). Nitrogen-rich diaminotriazine-based porous organic polymers for small gas storage and selective uptake. Polym. Chem..

[29] Ren YY (2018). Nitrogen-rich porous polyaminal network as a platform for iodine adsorption through physical and chemical interaction. J. Appl. Polym. Sci..

[30] Schwab MG (2009). Catalyst-free preparation of melamine-based microporous polymer networks through Schiff base chemistry. J. Am. Chem. Soc..

[31] Weng JY, Xu YL, Song WC, Zhang YH (2016). Tuning the adsorption and fluorescence properties of aminal-linked porous organic polymers through N-heterocyclic group decoration. J. Polym. Sci. Part A Polym. Chem..

[32] Mohan A (2020). Silver nanoparticles impregnated pH-responsive nanohybrid system for the catalytic reduction of dyes. Microporous Mesoporous Mater..

[33] Sabri MA (2021). Simultaneous adsorption and reduction of cr(Vi) to cr(iii) in aqueous solution using nitrogen-rich aminal linked porous organic polymers. Sustainability.

[34] Ben T (2011). Gas storage in porous aromatic frameworks (PAFs). Energy Environ. Sci..

[35] Bhunia A (2016). A photoluminescent covalent triazine framework: CO_2_ adsorption, light-driven hydrogen evolution and sensing of nitroaromatics. J. Mater. Chem. A.

[36] Guo Z (2018). Amorphous porous organic polymers based on Schiff-base chemistry for highly efficient iodine capture. Chem. Asian J..

[37] Chen Y (2015). Synthesis of conjugated microporous polymer nanotubes with large surface areas as absorbents for iodine and CO_2_ uptake. J. Mater. Chem. A.

[38] Liao Y, Weber J, Mills BM, Ren Z, Faul CFJ (2016). Highly efficient and reversible iodine capture in hexaphenylbenzene-based conjugated microporous polymers. Macromolecules.

[39] Mathai CJ, Saravanan S, Anantharaman MR, Venkitachalam S, Jayalekshmi S (2002). Effect of iodine doping on the bandgap of plasma polymerized aniline thin films. J. Phys. D. Appl. Phys..

[40] Yang Y (2019). Insight into volatile iodine uptake properties of covalent organic frameworks with different conjugated structures. J. Solid State Chem..

[41] Pourebrahimi S, Pirooz M (2021). Reversible iodine vapor capture using bipyridine-based covalent triazine framework: Experimental and computational investigations. Chem. Eng. J. Adv..

[42] Properties PI, Action M (2020). crossm in infection control and *Staphylococcus aureus* ecolonization. Oral Oncol..

[43] Bigliardi PL (2017). Povidone iodine in wound healing: A review of current concepts and practices. Int. J. Surg..

[44] Borjihan Q (2020). Pyrrolidone-based polymers capable of reversible iodine capture for reuse in antibacterial applications. J. Hazard. Mater..

