# Supplementary material for: Triazine-based porous organic polymers for reversible capture of iodine and utilization in antibacterial application
Source: Sci Rep. 2022 Feb 16;12:2638. doi: 10.1038/s41598-022-06671-0 (PMC8850422; doi:10.1038/s41598-022-06671-0)
Supplement: Supplementary file 1 — Supplementary Information. [file 41598_2022_6671_MOESM1_ESM.doc]

Electronic Supporting Information

**Triazine-based porous organic polymers for reversible capture of iodine and utilization in antibacterial application**

Anandhu Mohan, Mohammad H. Al-Sayah, Abdelrahman Ahmed, Oussama M. El-Kadri^*^

Department of Biology, Chemistry, and Environmental Sciences, American University of Sharjah, PO Box 26666, Sharjah, United Arab Emirates

*Corresponding author, Tel:+97165152787
 Email address: [oelkadri@aus.edu](mailto:oelkadri@aus.edu)

**Table of Contents**

1. **Characterization**

- FTIR
- TGA
- Powder X-ray

## Comparison Table

##

## Iodine Adsorption Kinetics

- Rate equations
- Data fitting and results

1. **Adsorption Isotherms**
2. **Antibacterial Study**


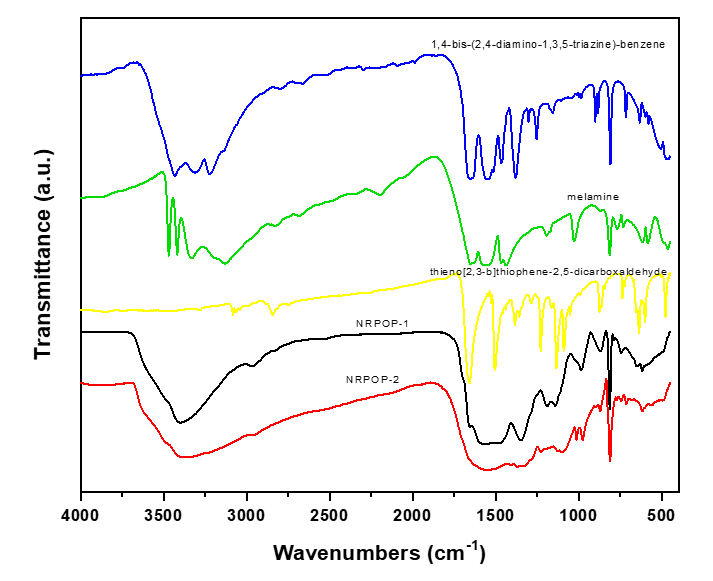


**Figure S1.** FTIR spectra of 1,4-bis-(2,4-diamino-1,3,5-triazine)-benzene, melamine, thieno[2,3-b]thiophene-2,5-dicarboxaldehyde, NRPOP-1, and NRPOP-2.


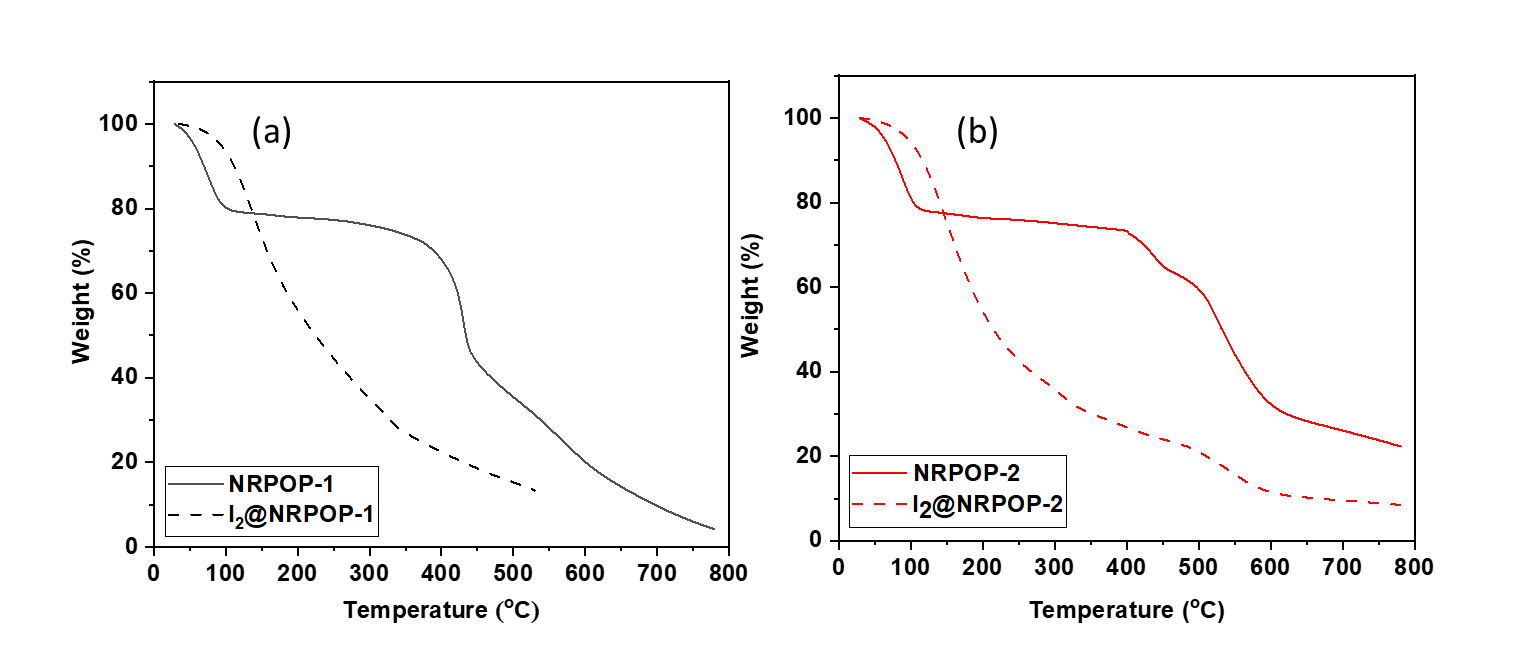


**Figure S2.** TGA curves of (a) NRPOP-1and I_2_@NRPOP-1 and (b) NRPOP-2 and I_2_@NRPOP-2.


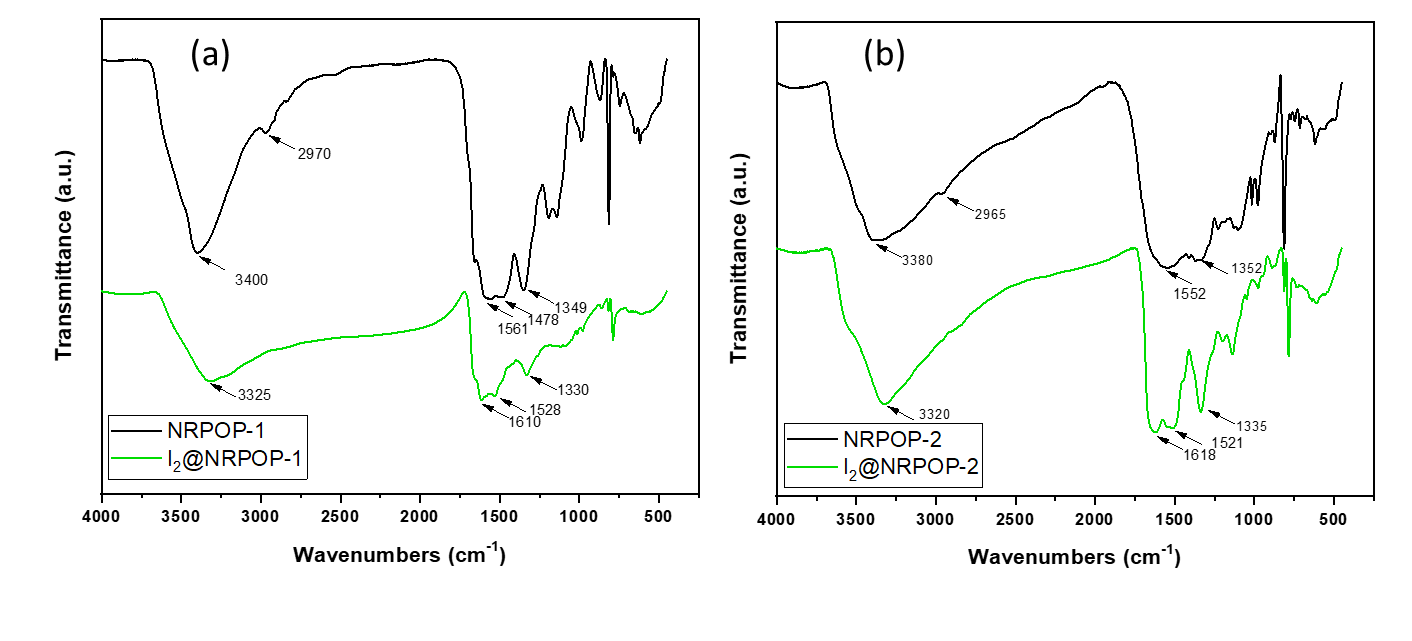


**Figure S3.** FTIR spectra of (a) NRPOP-1and I_2_@NRPOP-1 and (b) NRPOP-2 and I_2_@NRPOP-2.


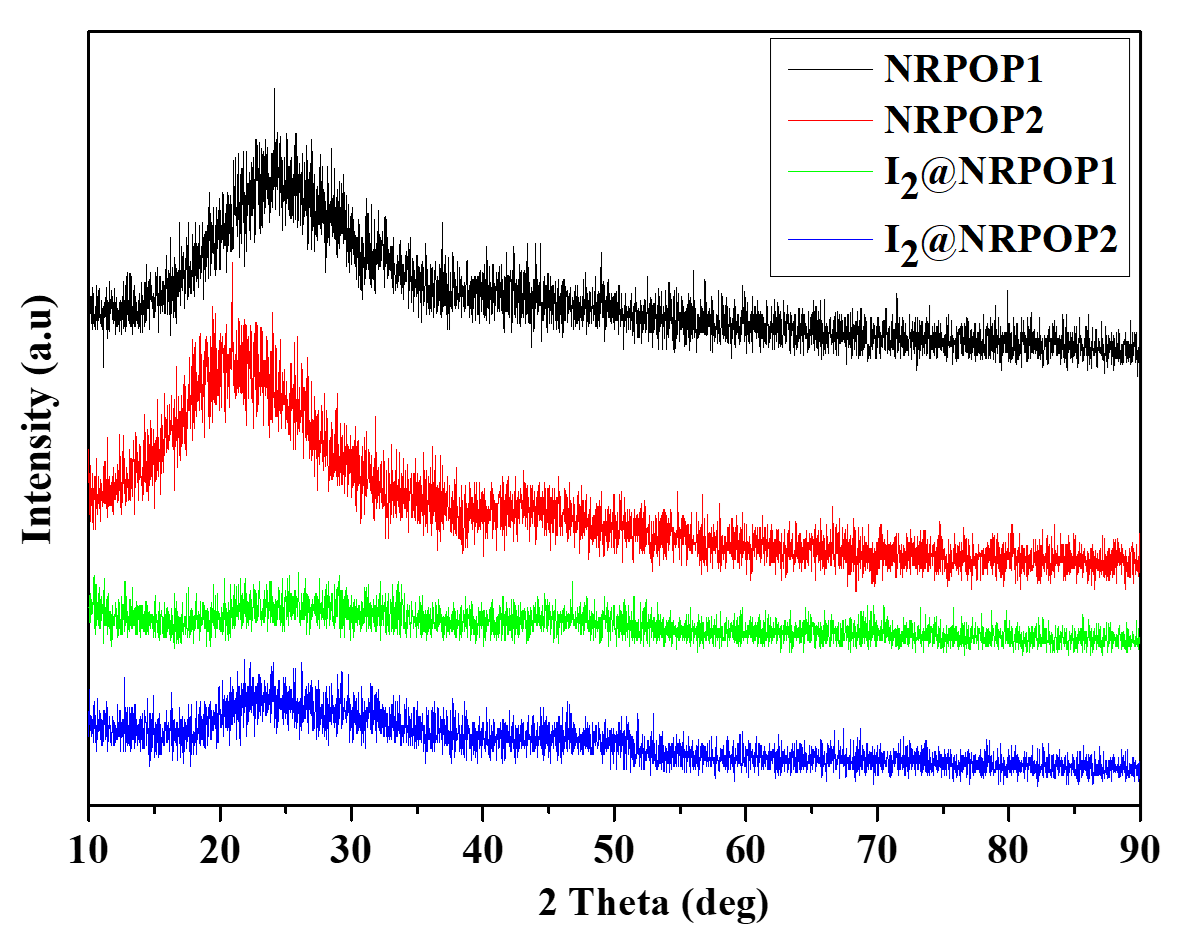


**Figure S4.** XRD patterns of NRPOP-1, NRPOP-2, I_2_@NRPOP-1, and I_2_@NRPOP-2.

**Table S1.** Comparison of the activities of the NRPOP-1 and NRPOP-2 with that of the previously reported adsorbent materials.

| **Number** | **Adsorbents** | **Temp**  **(K)** | **Pressure**  **(bar)** | **S_BET_**  **(m^2^ g**^-1^**)** | **Iodine uptake (Wt %)** | **Ref** |
| --- | --- | --- | --- | --- | --- | --- |
| 1 | **FCMP-600@4** | 350 | 1 | 636 | 141 | 1 |
| 2 | **NRAPOP-1** | 350 | 1 | 544 | 281 | 2 |
| 3 | **NRAPOP-2** | 350 | 1 | 424 | 271 | 2 |
| 4 | **PAOP-4** | 350 | 1 | 209.9 | 108 | 3 |
| 5 | **LHCP-3** | 348 | 1 | 1076 | 253 | 4 |
| 6 | **NRPP-2** | 350 | 1 | 1028 | 222 | 5 |
| 7 | **HCMP-3** | 358 | 1 | 82 | 316 | 6 |
| 8 | **CMPN-3** | 340 | 1 | 1368 | 208 | 7 |
| 9 | **CTF-CTTD-400** | 348 | 1 | 1684 | 357 | 8 |
| 10 | **CTF-CTTD-500** | 348 | 1 | 1334 | 387 | 8 |
| 11 | **SCMP-11** | 353 | 1 | 120 | 345 | 9 |
| 12 | **NRPOP-1** | 353 | 1 | 519 | 269 | This work |
| 13 | **NRPOP-2** | 353 | 1 | 456 | 317 | This work |

**Iodine Adsorption Kinetics**

The adsorption kinetics of iodine by the NRPOPs were examined at 25 °C by monitoring the removal of iodine from cyclohexane solution at 300 mg L^-1^. The obtained results are fitted with very good correlation to pseudo-second order kinetic model agreeing to equation II (below).

The equations of these kinetic models are:


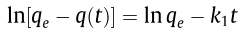
 (I) for pseudo-first order and


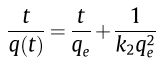
 (II) for pseudo-second order equation

Where, *q*_t_ is the amount of adsorbed iodine, *q*_e_ is the amount of adsorbed iodine at equilibrium, *k*_1_ is the pseudo-first order rate constant, *k*_2_ is the pseudo-second order rate constant, and *t* is the contact time. Table S2 is the kinetic parameters of pseudo-first order and pseudo-second order kinetic models.


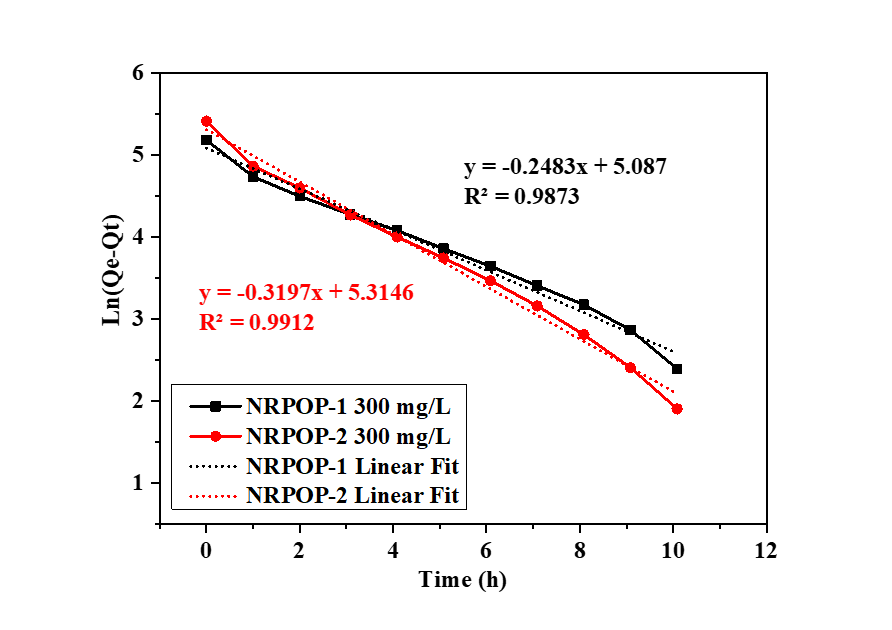


**Figure S5.** Pseudo first-order kinetics of iodine adsorption on NRPOP-1 and NRPOP-2 from iodine-cyclohexane solution. The points represent the experimental data at the concentration 300 mg L^-1^ and the solid lines represent the corresponding model fit (temperature: 298 K; adsorbent dosage: 1 mg mL^-1^).

**Table S2.** Parameters of pseudo first-order and second-order model fitting for iodine adsorption to the polymers in cyclohexane (300 mg L^-1^). *q*_e_ adsorbed amount at equilibrium (mg g^-1^), respectively, *k*_1_ and *k_2_* are the pseudo first-order and second-order constant of adsorption process (g mg^-1^ min^-1^).

|  | **Pseudo first-order model** | | | **Pseudo second-order model** | | |
| --- | --- | --- | --- | --- | --- | --- |
|  | *q*_e_ | *k*_1_ | R^2^ | *q*_e_ | *k*_2_ | R^2^ |
| **NRPOP-1** | 161.90 | 0. 2483 | 0.9873 | 227.27 | 0.00123 | 0.9956 |
| **NRPOP-2** | 203.28 | 0. 3197 | 0. 9912 | 270.27 | 0.00157 | 0.9999 |

**Adsorption isotherm**

**Langmuir and Freundlich isotherm**

Langmuir model was linearly fitted by plotting C_e_/Q_e_ vs Ce according to the following equation:

Where, C_e_ = the equilibrium concentration of adsorbate (mg L^-1^), Q_e_ = the amount of iodine per gram of the adsorbent at equilibrium (mg g^-1^). Q_m_ = maximum monolayer coverage capacity (mg g^-1^) K_l_ = Langmuir isotherm constant (L mg^-1^).

Freundlich isotherm model were linearly fitted by plotting (ln Q_e_) Vs (ln C_e_), according to Freundlich equation:

Where, K_f_ = Freundlich isotherm constant (mg g^-1^), N = adsorption intensity; C_e_ = the equilibrium concentration of adsorbate (mg L^-1^), Q_e_ = the amount of iodine adsorbed per gram of the adsorbent at equilibrium (mg g^-1^).


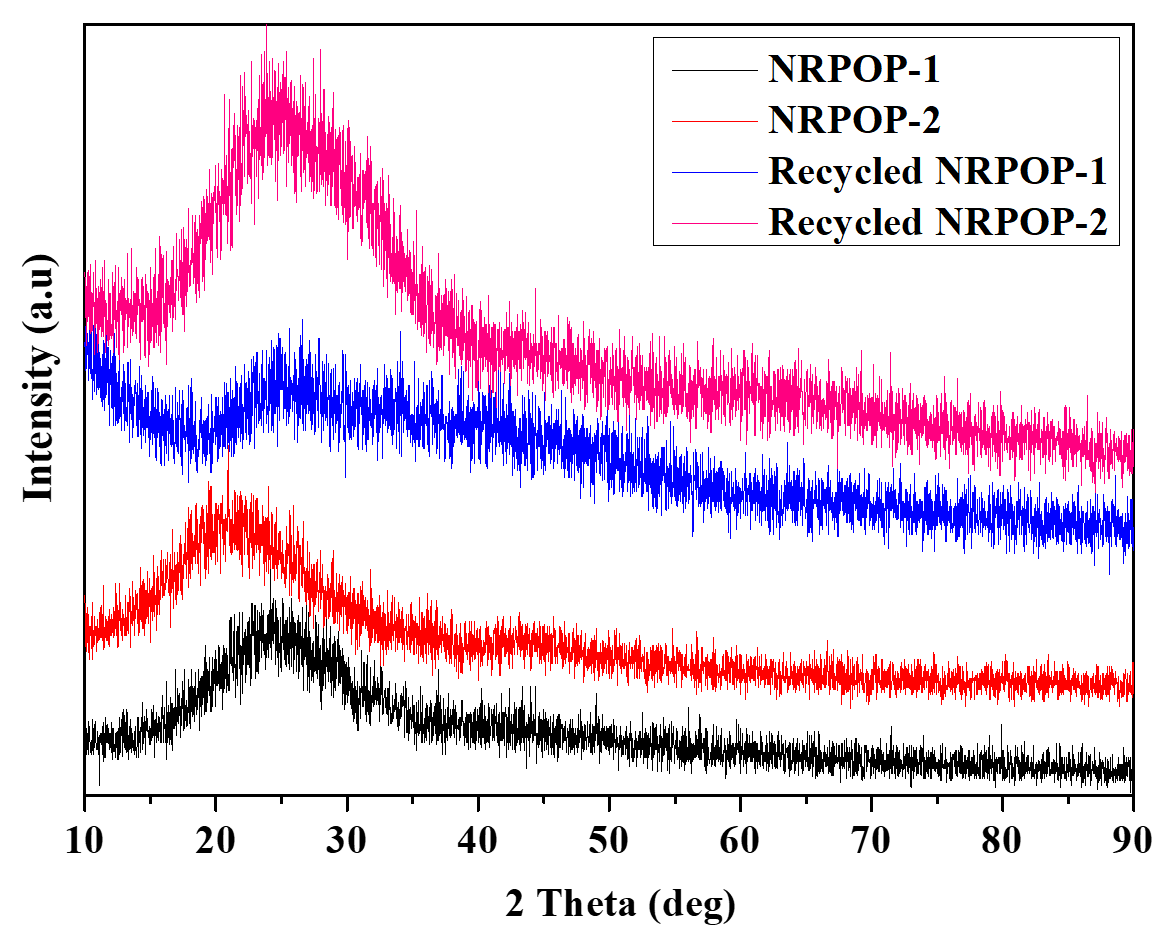


**Figure S6.** PXRD patterns of fresh and reused NRPOPs after the 4^th^ cycle.


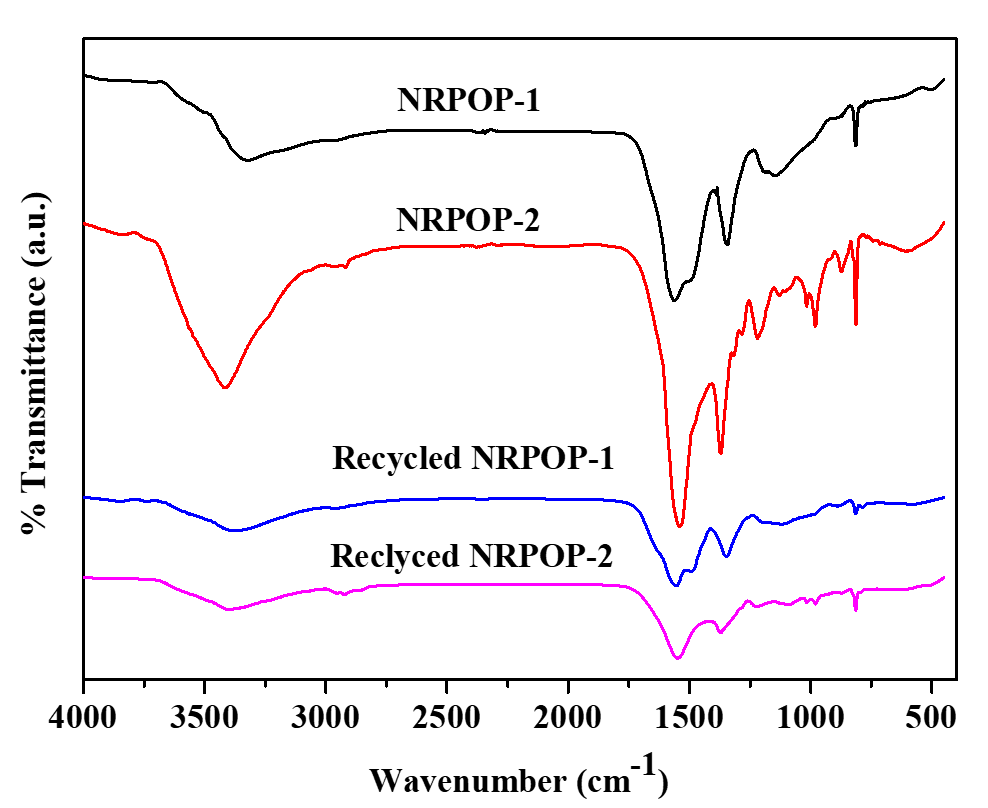


**Figure S7.** FTIR spectra of fresh and reused NRPOPs after the 4^th^ cycle.


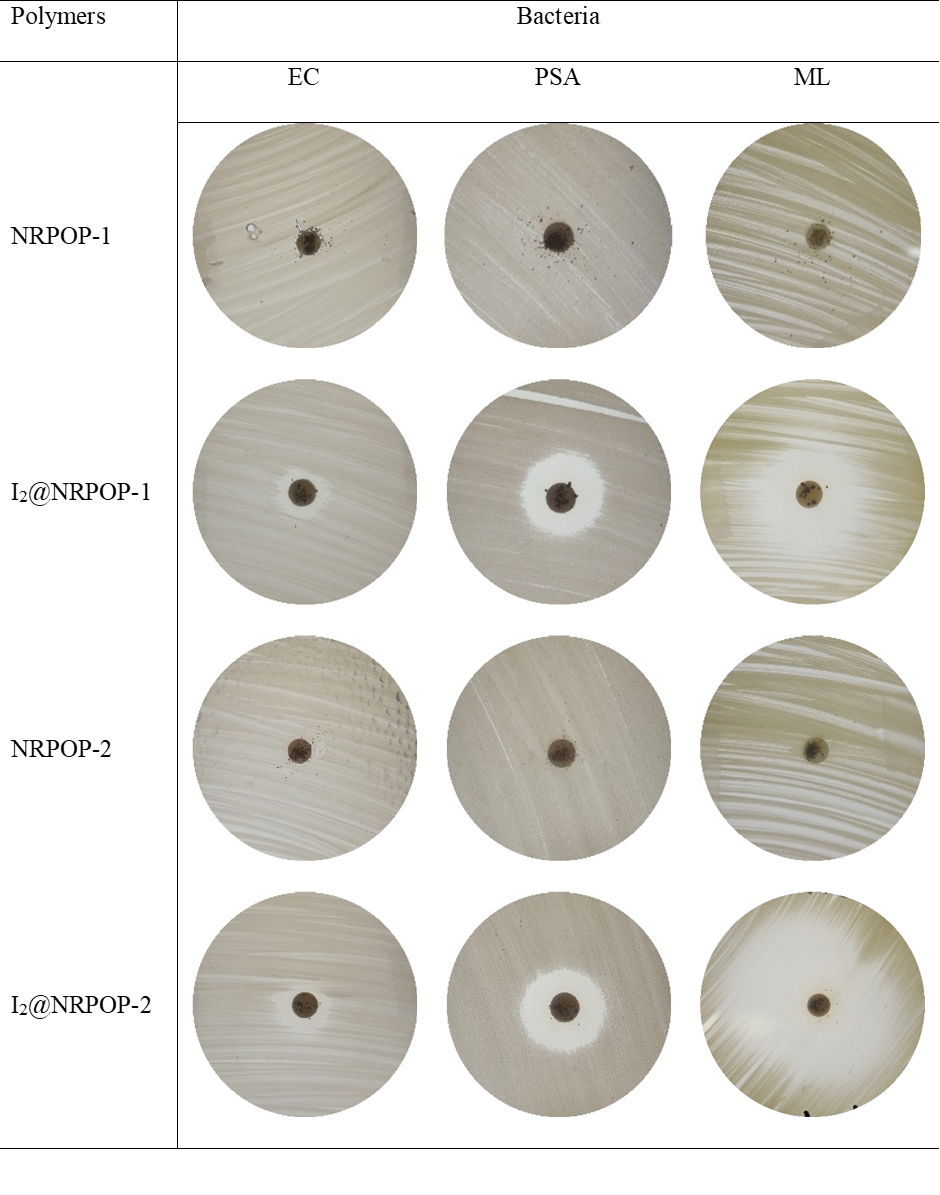


**Figure S8.** Photos of the Petri dishes in the presence of iodine-free and iodine-loaded polymers showing the bacterial growth and the bacterial inhibition zones.

**References**

1. Li, G., Yao, C., Wang, J. & Xu, Y. Synthesis of tunable porosity of fluorine-enriched porous organic polymer materials with excellent CO_2_, CH_4_ and iodine adsorption. *Sci. Rep.* **7**, 1–8 (2017).

2. Sen, S., Al-Sayah, M. H., Mohammed, M. S., Abu-Abdoun, I. I. & El-Kadri, O. M. Multifunctional nitrogen-rich aminal-linked luminescent porous organic polymers for iodine enrichment and selective detection of Fe^3+^ ions. *J. Mater. Sci.* **55**, 10896–10909 (2020).

3. Wang, Y. T. *et al.* Improving iodine adsorption performance of porous organic polymers by rational decoration with nitrogen heterocycle. *J. Appl. Polym. Sci.* **138**, 1–8 (2021).

4. Liu, N. *et al.* Construction of microporous lignin-based hypercross-linked polymers with high surface areas for enhanced iodine capture. *ACS Appl. Polym. Mater.* **3**, 2178–2188 (2021).

5. Abdelmoaty, Y. H., Tessema, T. D., Choudhury, F. A., El-Kadri, O. M. & El-Kaderi, H. M. Nitrogen-rich porous polymers for carbon dioxide and iodine sequestration for environmental remediation. *ACS Appl. Mater. Interfaces* **10**, 16049–16058 (2018).

6. Liao, Y., Weber, J., Mills, B. M., Ren, Z. & Faul, C. F. J. Highly efficient and reversible iodine capture in hexaphenylbenzene-based conjugated microporous polymers. *Macromolecules* **49**, 6322–6333 (2016).

7. Chen, Y. *et al.* Synthesis of conjugated microporous polymer nanotubes with large surface areas as absorbents for iodine and CO_2_ uptake. *J. Mater. Chem. A* **3**, 87–91 (2015).

8. Jiang, Q., Huang, H., Tang, Y., Zhang, Y., & Zhong, C*.* Highly porous covalent triazine frameworks for reversible iodine capture and efficient removal of dye. *Ind. Eng. Chem. Res.* **57**, 15114–1512 (2018).

9. Ren, F. et al. Novel thiophene-bearing conjugated microporous polymer honeycomb-like porous spheres with ultrahigh iodine uptake. *Chem. Commun.* **52**, 9797-9800 (2016).
